# Nanostructured Zn-Substituted Monetite Based Material Induces Higher Bone Regeneration Than Anorganic Bovine Bone and β-Tricalcium Phosphate in Vertical Augmentation Model in Rabbit Calvaria

**DOI:** 10.3390/nano12010143

**Published:** 2021-12-31

**Authors:** Lorena Benito-Garzón, Yasmina Guadilla, Idoia Díaz-Güemes, Iván Valdivia-Gandur, María-Cristina Manzanares, Arcadio García de Castro, Sussette Padilla

**Affiliations:** 1 Departamento de Anatomía e Histología Humanas, Facultad de Medicina, Universidad de Salamanca, 37007 Salamanca, Spain; 2 Departamento de Cirugía, Facultad de Medicina, Universidad de Salamanca, 37007 Salamanca, Spain; yguadilla@usal.es; 3 Centro de Cirugía de Mínima Invasión Jesús Usón, 10071 Cáceres, Spain; idoiadiaz@ccmijesususon.com; 4 Departamento Biomédico, Facultad de Ciencias de la Salud, Universidad de Antofagasta, Antofagasta 1270300, Chile; ivan.valdivia@uantof.cl; 5 Departamento de Anatomía y Embriología Humanas, Universidad de of Barcelona, 08007 Barcelona, Spain; mcmanzanares@ub.edu; 6 AzureBio S.L., Tres Cantos, 28760 Madrid, Spain; agc@qube.org (A.G.d.C.); spadilla@farm.ucm.es (S.P.); 7 Fundación QUBE, Tres Cantos, 28760 Madrid, Spain; 8 Departamento de Quimica en Ciencias Farmacéuticas, Facultad de Farmacia, Universidad Complutense de Madrid, 28040 Madrid, Spain

**Keywords:** monetite, zinc, hydroxyapatite, silica gel, amorphous calcium phosphate, vertical bone augmentation, bone regeneration, anorganic bovine bone, β-tricalcium phosphate

## Abstract

The capacity of a nanostructured multicomponent material composed of Zn-substituted monetite, amorphous calcium phosphate, hydroxyapatite and silica gel (MSi) to promote vertical bone augmentation was compared with anorganic bovine bone (ABB) and synthetic β-tricalcium phosphate (β-TCP). The relation between biological behavior and physicochemical properties of the materials was also studied. The in vivo study was conducted in a vertical bone augmentation model in rabbit calvaria for 10 weeks. Significant differences in the biological behavior of the materials were observed. MSi showed significantly higher bone regeneration (39%) than ABB and β-TCP (24%). The filled cylinder volume was similar in MSi (92%) and ABB (91%) and significantly lower in β-TCP (81%) implants. In addition, β-TCP showed the highest amount of non-osteointegrated particles (17%). MSi was superior to the control materials because it maintains the volume of the defect almost full, with the highest bone formation, the lowest number of remaining particles, which are almost fully osteointegrated and having the lowest amount of connective tissue. Besides, the bone formed was mature, with broad trabeculae, high vascularization and osteogenic activity. MSi resorbs gradually over time with an evident increment of the porosity and simultaneous colonization for vascularized new bone. In addition, the osteoinductive behavior of MSi material was evidenced.

## 1. Introduction

Teeth may be lost by dental disease, trauma, or as a consequence of a surgical procedure to resect part of a jaw pathologically damaged. Teeth also may be congenitally absent. After teeth loss, there is a lack of supporting bone due to atrophy, trauma, failure to develop, surgical resection, or infectious diseases, such as advanced periodontitis. This could be a problem for both implants and removable prostheses [1], which are getting loose by bone resorption.

Dental implants can only be implanted if there is enough healthy bone to stabilize them adequately. If not, bone augmentation is mandatory before implant placement. However, vertical bone augmentation is a major surgical clinic challenge and is not always achieved [2]. In particular, repairing the resorbed alveolar ridge in the edentulous posterior maxilla is one of the most critical challenges in modern dentistry [3]. The maxillary sinus, the nasal cavity, and the mandibular inferior alveolar nerve limit the bone height for proper implant placement. Several techniques are used for vertical bone augmentation, and usually, a high of 9 mm or more is necessary. Surgical procedures used for bone volume augmentation include guided bone regeneration, onlay or inlay grafting, ridge expansion and distraction osteogenesis. These techniques are usually associated with a donor site requirement, high complication rate, low success rate and inconsistent results [2,4].

For bone augmentation, it is necessary to use a fill material, like autogenous bone, allograft, xenograft, and synthetic bone grafts. Autogenous bone grafting remains the gold standard graft material. It is the most frequently used technique for vertical augmentation, despite its significant disadvantages, such as the requirement of bone harvesting and morbidity at the donor site, which includes pain, a potential infection, and loss of function [5,6]. A limited amount of intraoral bone makes harvesting and grafting challenging, and autologous bone grafts from other sites may not be suitable for dental implantation due to poor quality. In addition, the complications associated with autogenous iliac grafts are significant [7]. Attempts have been made to improve graft survival and volumetric maintenance. Calvarial bone has been reported to be superior to the iliac bone for onlay grafting because of decreased resorption, and it has been presented as a possible alternative method to achieve maxillary augmentation and sinus elevation [8]. Different biomaterials to replace bone autografts have been investigated in many animal and clinical studies [9,10,11,12,13,14,15,16] to overcome the mentioned bone harvesting drawbacks.

Bone augmentation procedures may be carried out before implant placement (two-stage procedure), or at the same time as implant placement (one-stage procedure), using different materials and techniques. An additional surgical episode is needed when carried out before placement, and the area is left to heal for some time before the implants are placed. Altogether, the ideal treatment for vertical bone augmentation should be the least invasive as possible. Therefore, it should not include an additional surgery for bone harvesting, and a two-step procedure involving two surgeries should be avoided.

The surgical technique combined with biomaterials, including bone graft and membranes, must ensure the maintenance of augmented bone volume over time. For this purpose, bone graft materials and membranes should become an effective alternative to autologous bone. They should be replaced by new bone, ensuring a high bone volume augmentation and conservation over time.

Allogenic bone from donor patients or xenograft materials has been used as an alternative to autologous bone. The most used xenograft material in implantology is an organic bovine bone (ABB). It is composed of hydroxyapatite (HAp), the mineral part of the bone. ABB is considered a very slow resorbed material that provides long-term volume preservation of augmented ridge [17,18,19,20]. However, non-resorbable or very slowly resorbable materials compromise the ultimate goal of complete regeneration of bone defects and may interfere with the integration of endosseous implants [21]. More resorbable synthetic calcium phosphates, such as β-tricalcium phosphate (β-TCP) are also frequently used in the clinic. Still, it is often associated with a loss of bone defect volume [22]. To assurance long-term preservation, materials combining HAp and TCP with different ratios have demonstrated good osteoconductivity and volume-maintaining capacity [23]. Bioactive glasses, usually composed of SiO_2_, Na_2_O, CaO and P_2_O_5_ have also been used as bone graft substitutes [24]. These materials are distinguished by their capacity of inducing new bone formation. Their resorption times depend on their composition.

Recently we have developed a new family of nanostructured materials combining calcium phosphates with different resorption rates: monetite, amorphous calcium phosphate and hydroxyapatite with silica gel [25,26,27]. These biomaterials were designed for acting like a temporary scaffold for bone growth, being gradually replaced by the newly formed bone and providing a local enrichment environment with ionic species, such as calcium, silica, phosphorus that favors bone regeneration. They have chemical and physical characteristics resembling the mineral part of the bone, such as nanostructure, composition, high specific surface area and porosity. The influence of HAp/Monetite ratio to achieve the best performance in bone regeneration was studied [26].

Zinc has been reported to induce osteoblastogenesis, osteoblast mineralization and to inhibit osteoclastic activity [28,29,30,31,32,33,34,35]. Therefore, Zn was incorporated in the material by partially substituting calcium in the monetite (MSi) [27].

This material effectively regenerates critical bone defects in sheep with significant resorption and replacement by new active and mature bone, both outside and inside granulates, with a high degree of vascularization and abundant osteogenic activity. It was also demonstrated that these materials induce bone formation and vascularization [26].

In addition, the material porosity increases after implantation, giving rise to a pore size compatible with vessel ingrowth [36]. Previous in vivo study in critical defects in sheep showed that MSi effectively restored bone volume and regenerated the defect being simultaneously replaced by new active bone [27]. In addition, the influence of HAp/Monetite ratio to promote bone formation was studied [26].

This work aimed to evaluate the vertical bone augmentation and bone regeneration capacity of a novel biomaterial (MSi) composed of Zn-substituted monetite, amorphous calcium phosphate, tricalcium phosphate and hydrated silica gel compared with two widely used bone graft materials, ABB and β-TCP. An osteoconductive model in titanium cylinders implanted in rabbit calvaria after 10 weeks was conducted. Their bone regeneration capacity and replacement by new osseous tissue were evaluated.

## 2. Materials and Methods

### 2.1. Studied Biomaterials

Three commercial granules were evaluated. Anorganic Bovine Bone ABB (Bio-Oss^®^) from Geistlich Pharma AG, Wolhusen, Switzerland, β-TCP (Cerasorb^®^) from Curasan AG, Kleinostheim, Germany and MSi (Sil-Oss^®^) from Azurebio SL, Madrid, Spain. ABB and MSi granulates were available with sizes between 0.25–1 mm and β-TCP granulates ranged between 0.5–1 mm.

ABB is obtained by protein extraction from bovine bone, and the β-TCP is a synthetic material produced by sintering. On the other hand, MSi is synthesized by a low-temperature process involving a hydraulic cementation reaction as previously described [25,26,27].

### 2.2. Biomaterials Characterisation

Materials were characterized as received by X-ray powder diffraction (XRD), scanning electron microscopy (SEM), N_2_ adsorption porosimetry and Hg porosimetry.

XRD patterns were recorded using a D8 Advance (Bruker AXS, Billerica, MA, USA) X-Ray diffractometer with LinxEye Super Speed Detector. The recording conditions were 10–70° (2θ), step size 0.05° (2θ), step time 1.05 s, λCuKα1 = 1.54056 Å, Va = 40 kV and Ia = 40 mA.

Biomaterials surface microstructure was examined on gold-coated samples using a TM-1000 table microscope (Hitachi Hi-Tech, Tokyo, Japan).

Specific surface area measurements were performed in a Monosorb Surface Area Analyser by N_2_ adsorption at −196 °C using the one-point BET method (MS-13, Quantachrome Instruments, Boynton Beach, FL, USA). Open porosity was determined in a Hg-intrusion porosimeter in the pore diameter range of 300 to 0.007 µm (PoreMaster 33, Quantachrome Instruments, Boynton Beach, FL, USA).

### 2.3. Experimental Animal Model

The animal experimentation was performed according to the national guidelines and conducted following the Spanish law (RD53/2013). Besides, the animal study was approved by the ethical committee for animal experiments of the Centro de Cirugía Minima Invasiva Jesus Usón (035/11) (Caceres, Spain) where in vivo experiments were carried out. Moreover, experiments were conducted according to European Communities Council Directive (2010/63/EU) and measures were taken to minimize pain or discomfort and animal suffering.

Eight New Zealand female rabbits aged between 8 and 9 months weighing 4.6 ± 0.2 Kg were premedicated with 0.25 mg/Kg intravenous midazolam (Midazolam Normon, Laboratorios Normon, Madrid, Spain) via the marginal ear vein. Then anesthesia was induced with 1 to 1.5 mg/Kg intravenous alfaxalone (Alfaxan, Jurox Limited, Dublin, Ireland). Orotracheal intubation was performed, and animals were connected to a ventilator. The anesthetized state was maintained with sevoflurane (Sevorane, Abbott Laboratories, Madrid, Spain) in oxygen (FiO_2_ = 1) at 3 L/min initial fresh gas flow rate (FGF) until 3–3.5% of sevoflurane in the expelled gas was reached followed by 0.5 L/min FGF. Mechanical ventilation was set at 20–25 rpm with a volume of 5–10 mL/Kg until normal values (35–40 mm Hg) were reached. Intravenous analgesia was carried out by ketorolac (Toradol, Atnahs Pharma Netherlands, Kovenhavn, Denmark) (1.75 mg/Kg) and tramadol (Adolonta inyec., Grünenthal Pharma, Madrid, Spain) (3 mg/Kg). Throughout the surgery, animals were infused intravenously with 5 mL/Kg/h of 0.9% saline. Heartbeat, hemoglobin saturation and respiratory frequency were monitored.

**Figure 1:**
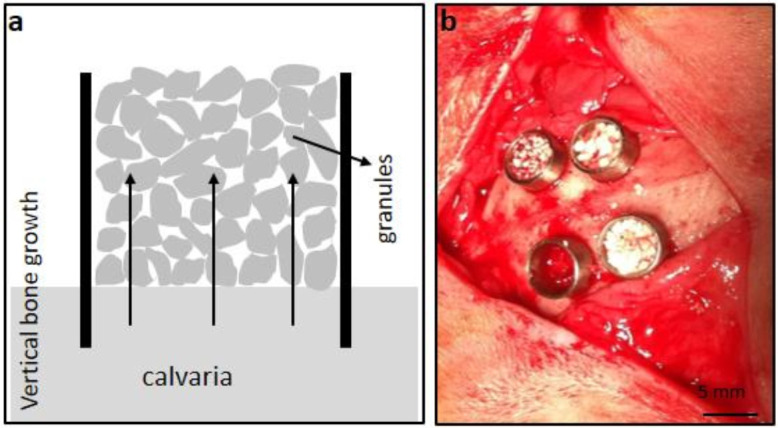
(**a**) schematic representation of vertical bone augmentation model using titanium rings in rabbit calvaria. (**b**) Four titanium rings (4 mm-height and 5 mm-diameter) were implanted in the rabbit calvaria filled with the studied granules.

Animals were placed in sternal recumbency, the head was shaved, and the cutaneous surface was disinfected with a povidone iodine solution. Calvaria bone was exposed through a 5 cm skin incision over the media line. Four circular osteotomies, 2 mm in depth, were carried out in the parietal bone for each animal, two anterior and two posterior at each side of the median sagittal suture. A 6 mm diameter and 0.5 mm thick trephine and a slow-speed electric handpiece with irrigation were used. Cortical bone was removed using a 3 mm surgical mono bevel chisel. Sterile grade 2 (ASTM B438) titanium rings with an internal diameter of 5 mm, 0.5 mm thick and 4 mm high, custom made by Lowde Titanium (Mallorca, Spain), were placed in each bone defects (Figure 1). MSi, β-TCP and ABB granules were randomly packed into the fitted cylinders up to the rim, and a blind code was assigned to each location. Subcutaneous tissues were closed with resorbable suture 3/0, and skin was relocated with 3/0 silk suture. Postoperatively, buprenorphine (Bupac, Richter Pharma, Wels, Austria) (0.004 mg/Kg/12 h) and metoclopramide (Metroclopramida Kerpharma, Terrasa, Barcelona, Spain) (0.5 mg/Kg/12 h) were administered subcutaneously as an analgesic for 3 days. Anti-inflammatory and antibiotic treatments with carprofen (Rimadyl, Zoetis, Madrid, Spain) (2 mg/Kg/12 h) and enrofloxacin (Baytril, Bayer Animal Health, Leverkusen, Germany) (7 mg/Kg/24 h), were administered subcutaneously for 7 days.

After general anesthetic by potassium chloride intravenous injection, animals were sacrificed at 10 weeks after surgery. Explanted samples, including implanted cylinder and underlying cranial bone, were obtained with an axe saw fitted with a carborundum disc. Samples were individually stored in 70° ethanol and code labeled.

### 2.4. Micro-CAT Scan

Micro Computer Aided Tomography (Micro-CAT) was carried out before the histological processing to determine the bone volume formed within the rings. It was performed on explanted samples by Trabeculae S.L (Orense, Spain) using an X-ray microtomography SkyScan 1172 (Bruker microCT NV, Kontich, Belgium). The parameters for the measurements were adjusted in order to minimize the strong X-ray absorption for titanium: the voltage was set to 100 KV, intensity to 100 µA, nominal resolution 10 µm, sample rotating angle 0.4°, frame averaging of 4, random movement of 25, rotation angle of 360° and filters of Al + Cu. Each sample was scanned for 5 h. The obtained tomograms were reconstructed using the Feldkamp algorithm modified in the application NRecon version 1.6.1.7 (Bruker microCT, Kontich, Belgium). Ring artifacts reduction of 20 and a beam hardening correction of 20 were applied.

### 2.5. Histological Evaluation

After micro-CAT analysis, the explanted samples were gradually dehydrated in ethanol solutions of increasing concentrations ranging from 70 to 100%. After dehydration, the samples were embedded in methyl methacrylate resin.

Samples were subjected to nondecalcified ground sectioning. The obtained blocks were cut with a low-speed saw microtome (Isomet, Bueher^®^ lake bluff, Dusseldorf, Germany) parallel to the titanium cylinder axis to get central sections. Some sections were stained directly with Stevenel’s Blue and van Gieson’s picro-fuchsin that stains mineralized bone in red, non-mineralized organic matrix in blue and biomaterial in grey [37]. The histological examination was carried out with a light microscope Nikon eclipse 90i fitted with a camera Nikon digital Sight DS-SMc (Nikon Instruments Inc., Melville, NY, USA).

### 2.6. Backscattering Electron Microscopy (BS-SEM)

Some sections of cylinders were photographed using backscatter electron microscopy (BS-SEM) and INCA software (version 1.1.0.34) (Oxford instruments, Abingdon, United Kingdom). BS-SEM imaging was employed to highlight the contrast among the resin, bone, and biomaterials. Usually, resin appeared black, bone was grey, and the biomaterial had a brighter contrast than bone.

### 2.7. Histomorphometric Analysis

The top of the implant, generally free of bone, showed density artifacts caused by Ti, therefore, 0.5 mm under cylinder top were discarded for micro-CAT analysis. X-ray absorption of Ti cylinder is very high; thus, its edges were observed poorly defined and blurred. This would cause an overestimation of bone during the binarization of images. In addition, it is known the osteoconductive behavior of Ti and bone growth through the inner cylinder surface was observed. Therefore, to avoid overestimation in the quantification of bone formed, 0.2 mm from the inner walls of the Ti cylinder inwards was discarded.

Therefore, to avoid overestimation in the quantification of the bone formed, 0.2 mm from the inner walls of the Ti cylinder inwards was discarded.

Thus, the total volume or volume of interest was considered as a cylinder of variable height depending on the depth to which the cylinder was implanted, with the upper limit 0.5 mm below the top edge of the cylinder with a 4.7 mm diameter inside the implant. Calvaria surface lines were analyzed to discard the previously existing bone and define the height at which the cylinder was implanted. Total volume includes new bone (NB), residual material (RM), intertrabecular spaces and connective tissue (CT). As occupied cylinder volume (OV) was considered the volume where the mineralized matrix was present also including intertrabecular spaces and residual material. Percentages of OV were calculated relative to total volume.

Percentages of new bone (% NB) and remaining material (% osteointegrated or % non-osteointegrated material) were calculated from the histological images. These analyses were carried out on longitudinal histology central sections of the defect. ImageJ software (version 1.49) was used for the histomorphometric study.

Statistical analysis was carried out by non-parametric two-tailed Mann-Whitney U Test, for a level of significance ≤ 95% (α ≤ 0.05). Percentages are presented as averages ± standard deviation (AVG ± SD) (see Table 1).

## 3. Results

### 3.1. Material Characterisation

Studied biomaterials showed different compositions, morphology, porosity and specific surface area.

#### 3.1.1. Composition

**Figure 2:**
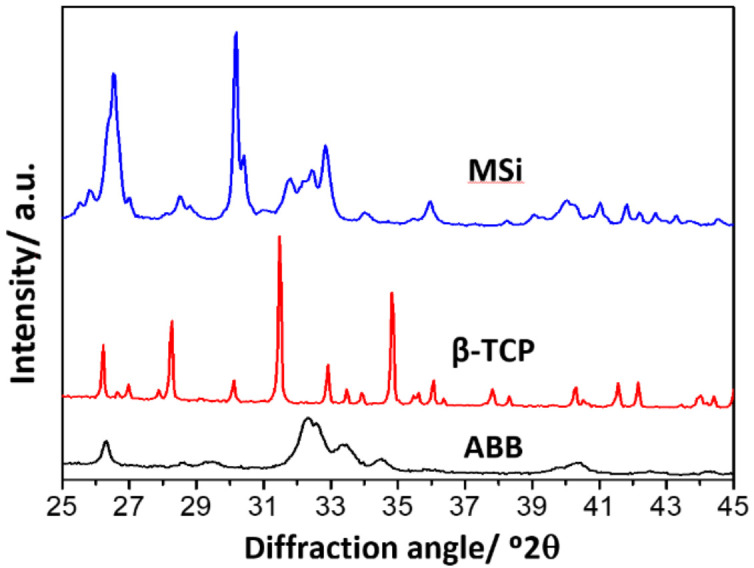
XRD pattern of studied materials. ABB is composed of low-crystallinity hydroxyapatite and β-TCP of pure β-tricalcium phosphate. MSi contains Zn-substituted monetite and hydroxyapatite as crystalline phases. It also contains amorphous calcium phosphate and hydrated silica gel [27].

ABB is composed only of low-crystallinity hydroxyapatite, characteristic of biological hydroxyapatite (Figure 2). β-TCP is also a single-component material consisting of β-tricalcium phosphate with high crystallinity (Figure 2).

On the contrary, MSi is a multicomponent material, showing XRD maxima corresponding to monetite and hydroxyapatite (Figure 2). A previous work [27] demonstrated that calcium in the monetite is partially substituted by Zn at 4 atom %, corresponding to 1.1 wt%. The incorporation of Zn caused shrinkage in the parameters and volume of the triclinic monetite cell unit and reduced the crystal domain size significantly compared with the material without Zn. The MSi composition is Zn-substituted monetite (57 wt%), hydroxyapatite (25 wt%), amorphous calcium phosphate (11 wt%) and hydrated silica gel (7 wt%).

#### 3.1.2. Microstructure, Porosity and Specific Surface Area

**Figure 3:**
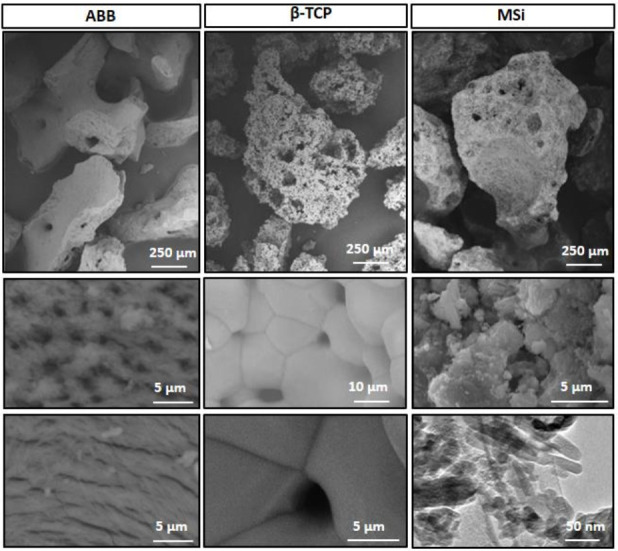
SEM micrograph of the studied materials. Granules had irregular shapes with sharper edges in ABB and smooth edges in β-TCP and MSi. ABB particles showed a porous surface with two pore morphologies. β-TCP showed the typical morphology of sintered materials with pores between grain boundaries. MSi showed a rough surface formed by agglomerates of similar porous morphology. These agglomerates are formed by homogeneously distributed nanometric particles, as observed in TEM images at the bottom right of the figure.

**Figure 4:**
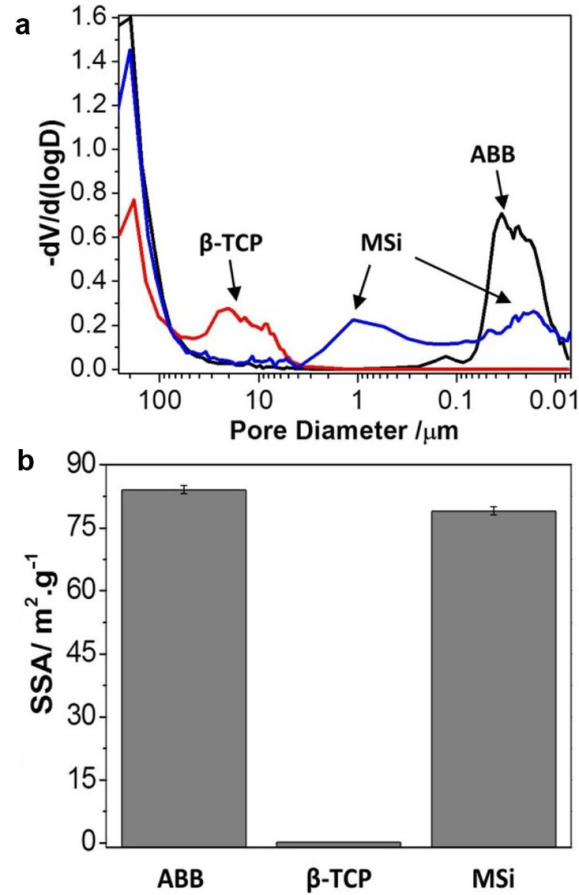
(**a**) Granules pore size distribution. ABB and MSi showed a similar total porosity but with different pore size distribution. ABB only showed nanometric pores whereas MSi had pores in a wider range, from micro to nanopores. β-TCP had the lowest porosity with the largest pore size, without pores in the meso or nanometric range. (**b**) ABB and MSi show a high specific surface area (SSA) of 80 m^2^/g whereas for β-TCP it is very low.

ABB granulates had an irregular shape with sharp edges. The material microstructure is characterized by elongated fibers and micropores through the material surface (Figure 3). These pores showed two morphologies: rounded of 1.6 μm diameter and the elongated ones of an average size of 6 μm long and 0.34 μm high (Figure 3). According to mercury porosimetry, the interconnection between these pores is smaller, with pores ranging between 200–7 nm, with a maximum centered at 40 nm (Figure 4a). The fibrous microstructure and the micro and nanopores observed by SEM and Hg porosimetry are due to spaces left by the collagen fibers during the deproteinization process of the treated bone [28,29,30,31,32,33,34,35,36,37,38,39]. Besides, some isolated macropores with sizes ranging between 130–20 μm (average size: 50 μm) were also observed by SEM, corresponding to spaces left by the Haversian blood vessel systems.

β-TCP granules also have irregular shapes but smooth edges and a porous microstructure. β-TCP shows a characteristic morphology of sintered materials with grains of high density and smooth surface (Figure 3). In addition, SEM revealed pores between 130–50 μm at the material surface with an average size of 70 μm (Figure 3). β-TCP has the largest intragranular pore ranging between 30–3 μm, with a maximum of 20 μm but does not have pores lower than 3 μm (Figure 4a). The high interconnected porosity of this material is due to the remaining pores between grains after sintering.

MSi presented a homogeneous and intimate distribution of its different components of nanometric size (Figure 3) as stated in previous work [27]. This material also showed irregular shapes with smooth edges and a rough, porous surface (Figure 3). SEM revealed the presence of macropores and mesopores both superficially and inside of granulates between 215–15 µm (average size: 53 µm) (Figure 3). Granules surface is formed by homogeneously distributed rounded agglomerates of similar morphology. These agglomerates provide microporosity (3.5–0.15 µm) (Figure 4a) and roughness homogenously distributed throughout the surface and inside the material. MSi has smaller pores (10 μm to 7 nm) with two maxima centered at 1 µm and 20 nm (Figure 4a).

The three studied materials also showed pores, by Hg porosimetry, higher than 60 μm with maxima around 200 μm, corresponding to intergranular spaces left between neighboring granules after packing (Figure 4a).

According to the bulk density of the materials and the Hg porosimetry, the intergranular porosity of the three materials was similar (32 vol%). The intragranular porosity of ABB and MSi was also similar (48 vol%). However, β-TCP showed the lowest intragranular porosity being only 32 vol%.

ABB and MSi showed a high specific surface area (SSA) (86 and 80 m^2^/g, respectively) consistent with their porous structure. In contrast, β-TCP has a very low specific surface area (<1 m^2^/g) (Figure 4b) characteristic of the sintering process and to the absence of both micropores lower than 3 μm and nanopores.

### 3.2. In Vivo Evaluation

ABB, β-TCP and MSi granules, with different resorption rates, were implanted in titanium rings in a vertical bone augmentation model in rabbit calvaria to study their bone regeneration capacity.

No post-surgical complications occurred except in one implant of ABB and other of MSi, where necrosis was observed at the implanted site so were discarded. Another ABB cylinder was broken during histological processing.

For the statistical analysis, two samples (ABB and MSi) were discarded as they presented a totally different behavior from the rest of the samples, which showed a homogeneous behavior.

#### 3.2.1. Histomorphometric Analysis

**Figure 5:**
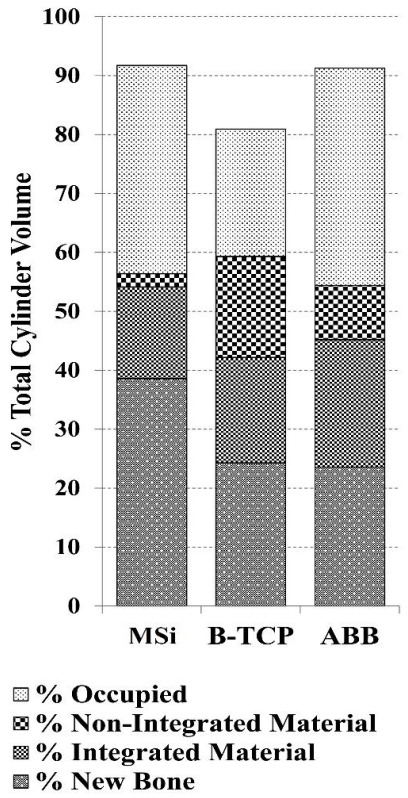
Graphical representation of the histomorphometric determinations for: ABB, β-TCP and MSi.

Comparative histomorphometric determinations and significance levels are shown in Table 1 and represented in Figure 5. After 10 weeks, ABB and MSi implanted cylinders achieved similar and the highest % of occupied space values. MSi also presented the highest % of new Bone and the lowest % of non-osteointegrated material values.

#### 3.2.2. ABB Granulates

The ABB group (*n* = 7) remained almost full and filled over 90 vol%. New bone was observed to vertically grow up to 55 vol% of the cylinder height. The area of the newly formed bone was 23.6 ± 7.3%. Osteointegrated ABB granules occupied 22 ± 10% of the cylinder area, whereas 9.2 ± 5.8% of the region remained with non-osteointegrated particles (Table 1 and Figure 5).

**Figure 6:**
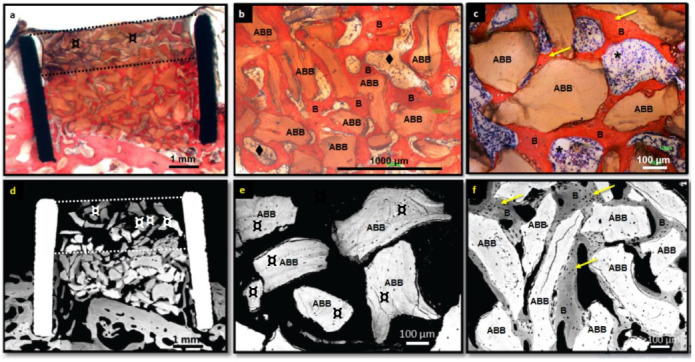
Histological (**a**–**c**) and BS-SEM images (**d**–**f**) of rings implanted with ABB granules. Rings remained almost full to the top of the cylinder (upper dotted line) with notable remaining granules of ABB (**a**,**d**). Non-osteointegrated granules were surrounded by connective tissue at the upper part of the cylinder (**a**,**d**,**e**). Vertical bone growth achieved the middle of the cylinder height (lower dotted line) (**a**,**d**). A very well osteointegrated particles surrounded by new bone were observed in all images, except e, which showed non-osteointegrated material present at the upper part of the cylinder. No changes in particle morphology after implantation were observed (**b**,**c**,**e**,**f**). Mature osteocytes were present at the newly formed bone (**c**,**f**). (Legend: B: newly formed bone; **¤**: non-osteointegrated granules; *: hematopoietic bone marrow; ⧫: adipose bone marrow; yellow arrows: osteocytes lacunae).

ABB showed a characteristic pattern of scarce new trabecular bone formed surrounding granules, which mainly appeared osteointegrated (Figure 6a–f). Due to the notable presence of ABB granules and its osteointegration, the new trabecular bone was formed in a thin, irregular and disorganized way and acquired the same sharp morphology as the granules. (Figure 6b,c,f). In the bone marrow spaces, adipose and predominating hematopoietic bone marrow were present (Figure 6b,c). Mature osteocytes were present in newly formed bone (Figure 6c,f). ABB granules did not show significant morphological changes after implantation (Figure 6b,c,e,f), and no signs of vascular or tissue invasion inside ABB granules were evident (Figure 6c,e,f). Non-integrated granules, presented at the cylinder upper part, appeared surrounded by connective tissue with no signs of invasion within granules (Figure 6a). The border between integrated and non-integrated material show continuity between newly formed bone and connective tissue surrounding the non-integrated granules (Figure 6a).

A homogeneous “activation” of calvaria bone was not possible during surgeries. This activation was intended to remove part of the outer cortical calvaria bone to encourage bleeding and the contact of biomaterials to the bloody bone. In the ABB group, calvaria bone was quite preserved in four implants, whereas it was greatly removed in the rest. Bone growth in the implants where the cortical bone was major conserved was 26 ± 6% and where calvaria was greatly removed 20 ± 8% (α ≤ 0.05). Therefore, conservation of the calvaria bone did not influence bone growth in the ABB group.

#### 3.2.3. β-TCP Granulates

**Figure 7:**
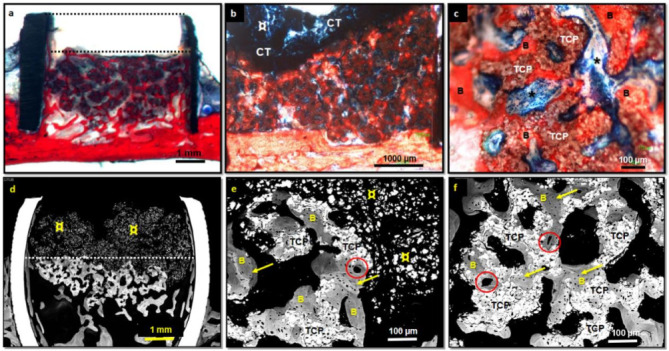
Histological (**a**–**c**) and BS-BSE images (**d**–**f**) of rings implanted with β-TCP granules. After implantation, 20% of the cylinder was empty (upper dotted line) (**a**). A high amount of remaining β -TCP was not osteointegrated (**b**,**d**,**e**) and vertical bone growth achieved only the middle of the cylinder height (lower dotted line) (**a**,**d**). Non-osteointegrated granulates were surrounded by connective tissue at the upper cylinder part (**b**). New trabecular formed bone showed homogeneous material osteointegration (**b**,**c**,**e**,**f**). Trabecular bone maturation was evident, with regular mineralized osteocytes lacunae and some vascular channels (**e**,**f**). Hematopoietic bone marrow was present (**c**). Legend: B: newly formed bone; ¤: non-osteointegrated material; *: hematopoietic bone marrow; yellow arrow: osteocytes lacunae; red circle: vascular channels; CT: connective tissue; TCP: β-TCP material.

Cylinders implanted with β-TCP maintained volume only up to 80%, the remaining 20% of the cylinder was empty after the study period (Figure 7a,d). In no case did the bone grow to the top of the cylinder or up to the occupied area but only to 41% of the cylinder height. The extent of new bone was 24 ± 15%. Osteointegrated β-TCP granules comprised 18 ± 12% of the cylinder area, and a high area remained occupied by non-osteointegrated β-TCP particles, whose size decreased considerably (Table 1 and Figure 5).

Significantly, the regeneration of implants containing β-TCP (*n* = 8) was heterogeneous, as can be inferred from the wide standard deviation of the parameters of the histomorphometric study.

In the β-TCP group, trabecular bone formation mainly occurred near the calvarial zone (Figure 7), where the β-TCP particles are homogeneously osteointegrated (Figure 7b,c,e,f). The newly formed bone presented a characteristic pattern with very thin trabeculae where the disintegrated particles interrupted their continuity (Figure 7c,e,f). The formed trabecular bone was mature, showing regular lacunae of mineralized osteocytes and some vascular channels (Figure 7e,f). Hematopoietic bone marrow was also observed (c). Large numbers of non-osteointegrated particles surrounded by connective tissue were observed (Figure 7b).

The transformation of the β-TCP granules was significant, from an initial size between 500 and 1000 µm to much smaller particle aggregates with diameters between 10–20 µm (Figure 7c,e,f).

Regarding the influence of the state of calvaria conservation in bone formation when β-TCP was implanted, cortical bone remained almost intact in three implants; in another case, only a small part of the cortical bone was removed. In the other four implants, most of the cortical were eliminated. In the first cases, scarce bone growth occurred (11 ± 9%), and a notable amount of non-osteointegrated material was observed. In the second case, 25% of bone was formed. The last group showed the highest bone growth (27± 11%) (α ≤ 0.05). Therefore, decortication of the calvaria bone influenced bone formation in the β-TCP group.

#### 3.2.4. MSi Granulates

The MSi group (*n* = 7) remained filled over 91%. New trabecular formed bone was observed to vertically grow up to 58 vol% of the cylinder height. MSi implants were characterized by high bone growth (39 ± 14%), the highest of the three studied materials, where the bone grows through the whole cylinder volume where particles were present. MSi osteointegrated particles were 15.6 ± 7.5% and non-osteointegrated 2.2 ± 5.6%, being the lower value of residual material of the studied granules (Table 1 and Figure 5).

**Figure 8:**
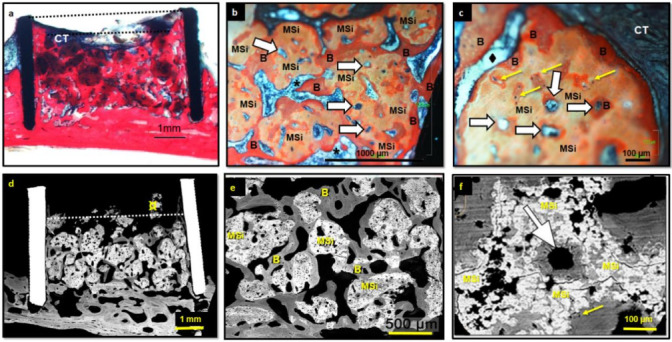
Histological (**a**–**c**) and BS-BSE images (**d**–**f**) of rings implanted with MSi. The volume of the cylinder remained almost completed filled (upper dotted line). The regenerated bone occupied the cylinder almost completely (lower dotted line) (**a**). The remaining particles were perfectly osteointegrated in a new trabecular bone (**a**–**f**). Granules became more porous and with a bigger pore size (**a**–**f**). New trabecular bone maturation was evident, with regular mineralized osteocytes lacunae and numerous vascular channels (**b**,**c**,**f**). Numerous vascular cavities were observed within osteointegrated MSi particles (**c**,**f**). Newly mineralized and cellularized bone was formed inside osteointegrated material granules (**c**,**f**). Both hematopoietic and adipose bone marrow were evident (**b**,**c**). Connective tissue was surrounded the scarce non-osteointegrated granulates at the top of the cylinder (**a**). (Legend: B: newly formed bone; ¤: non-osteointegrated material; *: hematopoietic bone marrow; ⧫: adipose bone marrow; yellow arrow: osteocytes lacunae; white arrow: vascular channels; CT: connective tissue).

MSi implants showed new trabecular bone, with well osteointegrated material and occupying almost the top of the cylinder (Figure 8a–f). New trabecular bone presented wide trabeculae with numerous vascular cavities and high osteogenic activity (Figure 8b,e). These 30–50 µm of diameter vascular channels were observed indistinctly inside the bone and in osteointegrated MSi particles (Figure 8b,c,e,f). MSi osteointegrated particles presented bone formation and cell bone colonization inside them (Figure 8c). A close relation between regenerated bone and osteointegrated MSi particles (Figure 8b,c,d,e) was evident, indicating their high osteoconduction and capacity to promote bone formation and maturation.

It should also be remarked that non-osteointegrated MSi particles showed bone formation inside them and on the surface that was in contact with connective tissue (Figure 8c). New trabecular bone showed osteocytes and notable vascular cavities (Figure 8b,c,f). Moreover, the development of both adipose and hematopoietic bone marrow was observed, a sign of bone maturation (Figure 8b,c).

MSi particles were reabsorbed both from the surface and the inside, increasing the porosity of material over time. These spaces have been penetrated by vascularized channels promoting new bone formation.

Concerning the conservation of calvaria bone in MSi implants, it was well preserved in five cases which showed 34 ± 13% of newly formed bone. In the other two MSi implants, cortical bone was almost eliminated and showed 37 ± 7% of new bone (α ≤ 0.05). Therefore, differences in the calvaria bone maintenance appeared not to influence bone growth in the MSi group, as has been observed in the ABB group.

## 4. Discussion

The present study compared the capacity of vertical bone regeneration of two established bone graph materials, ABB and β-TCP, with a novel synthetic nanostructured material composed of Zn-substituted monetite, amorphous calcium phosphate, hydroxyapatite and hydrated silica gel.

In oral surgery, bone regeneration procedures are often followed by endosseous implants. After the primary mechanical engagement, implant stability is highly dependent on the osteointegration achieved with bone regeneration and bone remodeling around the implant. Implant stability is the main parameter that determines implant loading schedule and treatment outcome [40,41,42,43,44].

The ideal bone graft to achieve vertical bone augmentation should be osteoconductive, i.e., act as three-dimensional support to guide bone growth, induce bone growth (osteoinductive) and be gradually resorbed and simultaneously replaced by newly formed bone [45,46,47,48]. The ultimate goal is to increase the necessary bone volume in the shortest possible time and completely replace the initial bone material with quality mature bone.

Vertical bone augmentation in rabbit calvaria is an established model for evaluating bone regeneration materials. The calvarial bone is ideal for vertical bone augmentation because of its embryological, morphological, and physiological similarities with the oral and maxillofacial region [49,50,51]. Besides, it has a large flat surface, facilitating the fixation of titanium rings [52].

One of the challenges of this model is that the graft material does not come out, so closed dome-shaped containers have been used [53]. In this case, the result may be affected by the osteoconductive effect of the chamber. To avoid this, a lid has been used instead of a closed chamber [54]. In the present study, the top of the cylinder was left open to make the model as similar as possible to medical applications, where part of the bone defect may be exposed to surrounding tissues or covered by a resorbable membrane.

Regarding methods used for histomorphometric analysis, micro-CAT scans provided a solid estimate of the radiopaque area, which was 90% for MSi, 89% for ABB and 80% for β-TCP. However, this technique could not discern between newly formed bone and residual material. Therefore, quantification of the other parameters was based on histology sections. Histomorphometric analysis coincided with the average estimations of the radiopaque area observed by micro-CAT scans.

In the histomorphometric evaluation of this work, %New Bone was restricted to areas within the cylinders that stained in red, by Van Gieson picrofucsine, excluding areas occupied by Residual Material and Connective Tissue. Although this criterion differs from some previous reports, where the Integrated Material was incorporated into the %New Bone value [55]. we consider it appropriate for a better-studied materials differentiation, considering their different resorption profiles.

Control materials, ABB and β-TCP, have an opposite in vivo behavior mostly due to their different composition and physical properties, such as porosity and surface specific area. ABB was considered as a control for a stable and no reabsorbable material. On the other hand, β-TCP was studied as an example of a resorbable material. Thus, we expected MSi might have an intermediate resorbable behavior compared to both controls (ABB and β-TCP).

Histological evaluation showed ABB implants were almost completely full, where material particles occupied almost the maxima height of the cylinder. New bone grew around particles, which presented good osteoconductive properties and osteointegration within the new bone formed. ABB could be considered as a stable material, with no sign of morphological changes over the implantation period, remaining practically unaltered. Almost 30% of the cylinder area remained occupied by ABB granules (21 ± 10% osteointegrated and 9 ± 6% non-osteointegrated, respectively). Present observations confirmed previous reports where ABB granules behave as osteoconductive scaffolds surrounded by fibrous connective tissue and replaced by new bone, with no invasion by connective tissue, no resorption nor cell colonization inside the granules [19,56]. This is supported by the fact that ABB granules are mostly insoluble and have intra-particle pores below 0.2 µm, averaging 0.03 µm [57], which are three orders of magnitude smaller than the 100 µm generally considered a requirement for cell colonization. [58]. This behavior was supported by the porosity measurements obtained in this work. Two different porosity morphologies were characterized: around (1.6 µm of diameter) and elongated (average size of 6 µm long and 0.34 µm high). Furthermore, mercury porosimetry results revealed the small size of interconnections between pores (from 200 to 7 nm). Although isolate macropores (20–130 µm) were observed by SEM, the average was 50 µm, so the requirement for cell colonization was not possible.

Concerning β-TCP, a synthetic pure-phase (>99%) used as control, its in vivo resorbable behavior was opposite to ABB. β-TCP granules changed their initial morphology from 500–1000 µm to particle agglomerates of very small size (around 15 µm) as a result of the dissolution of the contact points of sintered particles. Although both materials had opposite resorption, both showed similar bone formation: β-TCP (24 ± 15%) and ABB (24 ± 7%). β-TCP cylinder occupied area was the lowest (81 ± 9%) being the difference with ABB (91 ± 13%) and MSi (92 ± 3%) significant (α ≤ 0.05). This may be due to early resorption, not fulfilling the clinical requirements to space-maintenance function. β-TCP histological images confirmed that particles were osteointegrated after 10 weeks. In previous work, a small size of TCP granules (nanometers scale) induced higher porosity and larger specific surfaces, leading to an improved regenerative effect [59]. Previous comparative histology reports of implanted β-TCP granules showed they were resorbable and promoted new bone formation [60,61,62].

Nanostructured MSi studied material is composed of monetite, in which calcium was partially substituted by Zn, hydroxyapatite, amorphous calcium phosphate and silica gel. The experimental research was focused on a different approach to accelerate bone regeneration. The rationale behind MSi material composition was to provide a material capable of acting as a three-dimensional scaffold also accelerating bone formation by releasing ionic species, such as Ca, P, Si and Zn [25,26,27]. Thus, the main component, monetite, was chosen due to their higher resorption combined with amorphous calcium phosphate and hydroxyapatite with a slower resorption rate [26].

MSi implants’ in vivo behavior were characterized by a remarkable bone growth (38%), practically full-occupied cylinder area (91%) and most of the particles osteointegrated, being only 2% of the cylinder occupied by non-osteointegrated granules. The histomorphometric evaluation showed a significantly greater new bone for MSi (39 ± 14%) than for β-TCP (24 ± 15%) or ABB (24 ± 7%). The occupied space was very similar for ABB and MSi, but both were significantly different with the β-TCP group. Remarkable resorption promoting the bone formation of MSi was evidenced after 10 weeks of implantation.

Both histology and BS-SEM images revealed, unlike the controls, that MSi granules appeared mostly osteointegrated, with evident signs of cell colonization and new bone formation inside the particles. The release of Ca, Si, P and Zn ions to the environment [26,27], promote the formation of an apatite surface layer which is generally associated with improved osteointegration surface [63,64].

The initial intra-granular pore sizes of MSi (3.5–0.15 µm) are not large enough for cell colonization but allow the diffusion of the released species, favor the entry of proteins and nutrients and the reabsorption of granules. The biomaterial resorption provided larger pores adequate for neovascularization, followed by cell colonization. This process is essential to bone regeneration [38,65].

MSi is a nanostructured material, the same as bone apatite. Nanometric calcium phosphates have shown enhanced osteoblast adhesion, proliferation and mineralization compared with microstructured materials leading to increased formation of new bone tissue within a short period [66,67]. This is an important feature over microstructured materials, as nanometric morphology contributes to increased reactivity of the material, improved protein adsorption and interaction with osteoblasts, as well as facilitating bio-material passive and active resorption by osteoclast and macrophage activity [26,66,67,68,69,70].

In addition to composition and morphology, the biomaterial also showed relevant features that contribute to their biological performance as bone regeneration material, such as high interconnected porosity, specific surface area and surface roughness. These advantageous physical properties are related to the synthesis method which involves a hydraulic cementing reaction in which material consolidation is achieved through a low temperature dissolution–precipitation process, giving rise to hydrated compounds with morphologies and compositions very similar to the calcium phosphates found in the mineralized tissues, high specific surface area, porosity, surface roughness and nanostructure [26,27].

Monetite, a major component of MSi, has been previously reported as resorbable material that is gradually replaced by new bone, that grows inside and around grafted granules in the bone regeneration model in rabbit calvaria [71] or in alveolar bone human defects [72]. The presence of silicon in MSi could contribute to the initiation of calcified tissue mineralization [73]. Recent studies suggested that silicon has a dual nature in bone metabolism, with stimulatory effects on osteoblasts and inhibitory effects on osteoclasts [74]. Furthermore, MSi is also composed of zinc, which has been reported to contribute to the resorption of other calcium phosphates, such as β-TCP 35] and may also play an important role in the resorption of MSi. However, as for β-TCP, bovine HA or BCP (Biphasic calcium phosphate) the influence of material dissolution processes and osteoclast involvement remain unclear [75]. Our results showed that the calcified tissues studied at 10 weeks post-implantation, were more mature and structured in wider trabeculae in MSi group compared to the other materials. Furthermore, the remaining MSi particles showed several areas of neovascularization and new bone formation inside the particles, being a sign of a process of substitution of biomaterial by bone.

Regardless of the results obtained in this work, in other studies where ABB was grafted in titanium cylinders (9 mm of diameter) in rabbit calvaria for 4 weeks, bone volume was similar 13 [76] and 11% [19] respectively. However, in our work, ABB new bone was higher (23%), but a long period was evaluated (10 weeks) of bone formation over time. Related to the remaining ABB graft, similar values were obtained 35 [77] vs. 30% (21% osteointegrated + 9% non-osteointegrated) in our work, showing no resorption of ABB granules from 4 to 10 weeks of study. In other similar work [56], where titanium cylinders covered by a lid were studied for 12 weeks, ABB occupied cylinder volume was 94% vs. 91% obtained in the present study. Additionally, in other work using a closed cylinder (6 mm diameter and 1 mm height) at 6 weeks, bone values for BCP (Biphasic calcium phosphate HA/TCP) were slightly higher than for ABB (35 and 25% respectively) [77]. Bio-Oss^®^ new bone value was similar to our results (23%). In other work, where a granulated brushite-TCP cement was studied at 4 weeks [78], bone regeneration was 16% vs. 38% obtained for our MSi studied material. Besides, in a previous work, where a 3D α-TCP/HA material was studied compared to ABB and β-TCP in titanium cups at sheep calvaria at 8 and 16 weeks [79], new bone values at 8 weeks were lower (ABB 13%, β-TCP 13% and α-TCP/HA 23%) than compared to the obtained is our work (ABB 23%, β-TCP 24% and MSi 39%). At 16 weeks new bone occupied about 40% of the volume under the titanium hemispheres in the three tested materials [79], with the value being very similar to MSi (39%) in our work at 10 weeks. It should be considered that in our study open titanium cylinders were used, so the exposure to biological fluids was higher. In relation to degradation, the volume of the remaining material for ABB and α-TCP/HA were the same for 8 and 16 weeks (40 and 45% respectively) [79], mostly due to the presence of HA on their chemical composition. In our work, the remaining ABB was quite similar (31%). β-TCP remained material was 33% at 8 weeks [79], similar to β-TCP (34%) in our work, whereas for our studied material MSi was 17%, highlighting its higher resorption. In previous work, 3D printed monolithic monetite blocks were also studied in calvaria rabbits at 8 weeks [13]. Monetite blocks new bone was 43%, similar to MSi (39%) in our work. Dental implants can be successfully placed into regenerated bone with a bone volume of 30–40% [80]. There are several studies where bone augmentation procedures with dental implants have been stabilized with this amount of bone volume [81,82].

In the experimental model used in this work, the importance of decortication or cortical bone perforations is somewhat the subject of some controversy. Some reports supported that cortical preservation compromised results, leading to increased bone augmentation [83,84]. However, other authors concluded that the cortical bone plate was compromised at the site of the titanium cylinder and there may be sufficient damage to see no difference between the corticated and decorticated experimental setups [54]. This work selected decortication based on the typical clinical scenario where graft materials are implanted directly with bleeding bone. However, as explained before, decortication was not homogeneous in all samples, and it appeared to influence only the bone formation of the β-TCP group. Therefore, we suggested that the composition and physicochemical properties of the materials influence vertical growth more than the state of the calvaria bone. Thus, ABB and MSi materials could be considered to have a good osteoconductive capacity even if the calvaria bone has been preserved. However, β-TCP osteoconductive capacity was not good enough and needed to have an active bleeding bone to promote bone formation.

This study is limited to a small space, using titanium cylinders in rabbit calota, which is not representative of clinical practice. Therefore, the vertical bone augmentation capacity of this material needs to be further evaluated in clinical settings. Additional studies are also needed to investigate the osseointegration and stability of the endosseous implant when MSi is used.

Resorbable graft materials, such as MSi may increase implant stability by facilitating biological processes associated with bone remodeling during the bone regeneration period and promoting dense bone formation in the vicinity of the endosseous implant [85], avoiding interference of residual graft material.

## 5. Conclusions

The three evaluated materials were capable of vertical bone augmentation but with a very different behavior highly conditioned by their different composition, solubility, morphology, specific surface area, porosity and pore size distribution, and granular cohesion.

ABB maintained a high volume of the defect filled as it remains in the site without resorption. It shows a good osteoconductive behavior related to its high specific surface area and porosity in the micro and nanometric range.

β-TCP lost its granular structure and appeared disintegrated in small particles (15 µm) because of the reabsorption of the neck union between grains. In addition, the specific surface area of this material is extremely low, with no porosity inside the grains as characteristic of sintered materials. All these factors were detrimental for supporting bone growth and, therefore, volume preservation showing a significant lowest volume augmentation and a higher amount of non-osteointegrated material.

On the contrary, MSi showed the best bone regeneration and vertical augmentation performance. It achieved the highest amount of new bone, the lowest amount of connective tissue and almost all the remaining particles osseointegrated. New trabecular bone was mature, vascularized with high osteogenic activity formed by broad trabeculae.

MSi showed clear signs of resorption, bone ingrowth, cellular colonization and several vascular cavities outside and inside granules, being the remaining particles perfectly osseointegrated.

The very scarce MSi particles non-osteointegrated surrounded by connective tissue also showed bone formation and vascularization inside granulates and around it, indicating that material induces bone formation, i.e., osteoinductive.

MSi is a nanostructured multicomponent material, whose components have different reabsorption rate that gradually resorbs, and simultaneously releases ionic species that favor bone regeneration, such as Zn, Si, Ca and phosphate ions. The excellent biological behavior of this material is also related to its high specific surface area high roughness, high interconnected porosity with pores ranging from macro to nanopores, good granular cohesion, homogeneous and intimate distribution of its components and nanometric structure similar to bone apatite.

Decortication of the calvaria bone influenced bone formation in the β-TCP group, but not in ABB and MSi implants, indicating better osteoconductive behavior of the latter compared to β-TCP.

## Figures and Tables

**Table 1:** Histomorphometric determinations and Mann-Whitney significance test. Average ± standard deviations (SD) expressed as % of total cylinder volume for ABB, β-TCP and MSi. Significance was determined by the non-parametric two-tailed Mann-Whitney U test for the significance level: 90. (α ≤ 0.1), ≤95% (α ≤ 0.05) or ≤98% (α ≤ 0.02).

Average ± SD	% Occupied Space	% New Bone	% Integrated Material	% Non-IntegratedMaterial	% Connective Tissue
ABB (*n* = 7)	91 ± 13	23.6 ± 7.3	22 ± 10	9.2 ± 5.8	31 ± 10
β-TCP (*n* = 8)	80.9 ± 9.1	24 ± 15	18 ± 12	17 ± 18	35 ± 12
MSi (*n* = 7)	91.7 ± 2.9	39 ± 14	15.6 ± 7.5	2.2 ± 5.6	18 ± 11
Mann-Whitney (N.S: Non Significant)					
ABB vs. β -TCP	α ≤ 0.05	N.S	N.S	N.S	N.S
ABB vs. MSi	N.S	α ≤ 0.1	N.S	α ≤ 0.02	α ≤ 0.05
MSi vs. β –TCP	α ≤ 0.05	α ≤ 0.1	N.S	N.S	α ≤ 0.02

## Data Availability

The data is included in the main text.
